# Activation of the EGFR/PI3K/AKT pathway limits the efficacy of trametinib treatment in head and neck cancer

**DOI:** 10.1002/1878-0261.13500

**Published:** 2023-08-31

**Authors:** Ofra Novoplansky, Avital B. Shnerb, Divyasree Marripati, Sankar Jagadeeshan, Raghda Abu Shareb, Cristina Conde‐López, Jonathan Zorea, Manu Prasad, Talal Ben Lulu, Ksenia M. Yegodayev, Chen Benafsha, Yushi Li, Dexin Kong, Fengshen Kuo, Luc G. T. Morris, Ina Kurth, Jochen Hess, Moshe Elkabets

**Affiliations:** ^1^ The Shraga Segal Department of Microbiology, Immunology, and Genetics Ben‐Gurion University of the Negev Beer‐Sheva Israel; ^2^ Division of Radiooncology‐Radiobiology German Cancer Research Center (DKFZ) Heidelberg Germany; ^3^ Department of Chemical Engineering Ben‐Gurion University of the Negev Beer‐Sheva Israel; ^4^ Department of Biochemistry University of Oxford Oxford UK; ^5^ School of Pharmaceutical Sciences Tianjin Medical University Tianjin China; ^6^ Immunogenomics and Precision Oncology Platform, Department of Surgery Memorial Sloan Kettering Cancer Center New York New York USA; ^7^ Section Experimental and Translational Head and Neck Oncology, Department of Otolaryngology, Head and Neck Surgery University Hospital Heidelberg Germany; ^8^ Research Group Molecular Mechanisms of Head and Neck Tumors Deutsches Krebsforschungszentrum (DKFZ) Heidelberg Germany

**Keywords:** drug resistance, head and neck cancer, PI3K and EGFR signaling, Trametinib

## Abstract

Blocking the mitogen‐activated protein kinase (MAPK) pathway with the MEK1/2 inhibitor trametinib has produced promising results in patients with head and neck squamous cell carcinoma (HNSCC). In the current study, we showed that trametinib treatment leads to overexpression and activation of the epidermal growth factor receptor (EGFR) in HNSCC cell lines and patient‐derived xenografts. Knockdown of EGFR improved trametinib treatment efficacy both *in vitro* and *in vivo*. Mechanistically, we demonstrated that trametinib‐induced EGFR overexpression hyperactivates the phosphatidylinositol 3‐kinase (PI3K)/AKT pathway. *In vitro*, blocking the PI3K pathway with GDC‐0941 (pictilisib), or BYL719 (alpelisib), prevented AKT pathway hyperactivation and enhanced the efficacy of trametinib in a synergistic manner. *In vivo*, a combination of trametinib and BYL719 showed superior antitumor efficacy vs. the single agents, leading to tumor growth arrest. We confirmed our findings in a syngeneic murine head and neck cancer cell line *in vitro* and *in vivo*. Taken together, our findings show that trametinib treatment induces hyperactivation of EGFR/PI3K/AKT; thus, blocking of the EGFR/PI3K pathway is required to improve trametinib efficacy in HNSCC.

## Graphical abstract

MEK inhibition by trametinib leads to rapid hyperactivation and upregulation of YAP1. YAP1 overexpression and localization in the nucleus are associated with the upregulation of epidermal growth factor receptor (EGFR) transcription. Overactivation of EGFR induces phosphatidylinositol‐4,5‐bisphosphate 3‐kinase/protein‐kinase‐B (PI3K/AKT) pathway signaling, which limits treatment efficacy. The combination of MEK and PI3K inhibition prevents AKT hyperactivation, resulting in superior antitumor activity compared with monotherapy.

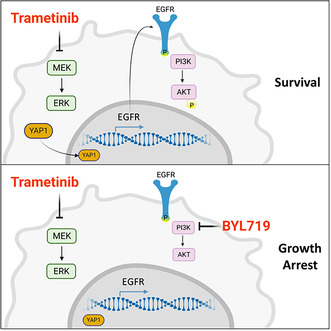

**Abbreviations**
4NQO: 4‐nitroquinoline 1‐oxide
BSA: bovine serum albumin
CMC: carboxymethylcellulose
DMSO: dimethyl sulfoxide
EGFR: epidermal growth factor receptor
HNSCC: head and neck squamous cell carcinoma
i.p.: intraperitoneal
MAPK: mitogen‐activated protein kinase
mTOR: mammalian target of rapamycin
PBS: phosphate‐buffered saline
PDX: patient‐derived xenograft
PI3K/AKT: phosphatidylinositol‐4,5‐bisphosphate 3‐kinase/protein kinase B
RTKs: receptor tyrosine kinases
TBS: Tris‐buffered saline
WT: wild type
YAP1: yes‐associated protein 1

## 1 Introduction

Head and neck squamous cell carcinoma (HNSCC) is a heterogeneous malignancy that arises from the squamous epithelium layer of the oral cavity, pharynx, and larynx [1]. Over 60% of HNSCC patients are diagnosed with stage III or IV cancer, which is characterized by a high risk of recurrence and a poor prognosis (5‐year overall survival < 50%) [2, 3]. Multimodality treatment comprising surgery, radiation, and chemotherapy is the standard of care for HNSCC patients [1]. Where comorbidities prevent the use of cytotoxic chemotherapy or when surgical resection is not possible, a targeted agent, such as the epithelial growth factor receptor (EGFR) monoclonal antibody, cetuximab, or the immune checkpoint inhibitors, pembrolizumab and nivolumab, may be used [1]. Despite the availability of these options, the prognosis for advanced‐stage HNSCC patients remains dire and calls for the identification of new targets and the development of new intervention strategies [4, 5].

One such target could be the mitogen‐activated protein kinase (MAPK) pathway, which is one of the best studied pathways in cancer due to its involvement in cell proliferation, survival, migration, and transformation [6]. The MAPK pathway can be activated by mutations in genes, such as RAS and RAF, or by overexpression of receptor tyrosine kinase (RTK) receptors, such as EGFR, or their ligands [7, 8]. In HNSCC patients, ~ 18% of tumors harbor mutations that affect MAPK pathway activation [9], and those tumors exhibit unique clinical, signaling, and immunological characteristics [10, 11, 12].

A promising cytotoxic drug targeting the MAPK pathway is trametinib, a second‐generation allosteric, ATP non‐competitive inhibitor designed to inhibit the MEK1 and MEK2 kinases in the MAPK pathway [13]. Trametinib has been approved for the treatment of melanoma, non‐small cell lung cancer, and thyroid cancer patients harboring the BRAF V600E mutation [14, 15, 16]. In line with the prediction that it would be effective in the treatment of high‐risk HNSCC [17], trametinib was tested in a phase II neoadjuvant window‐of‐opportunity clinical trial—with promising results [18]. However, a follow‐up study showed that tumors developed resistance to trametinib monotherapy via upregulation of yes‐associated protein 1 (YAP1) [19]. That study, and others that followed it, established the importance of the MAPK pathway in HNSCC and highlighted the clinical need to uncover the molecular mechanisms of resistance to trametinib with the aim to improve its efficacy in the treatment of patients with HNSCC [9, 10].

In the current study, we sought to uncover the signaling machinery that enables HNSCC tumor cells to overcome trametinib treatment. Our findings showed that exposure of HNSCC cell lines to trametinib‐induced upregulation and activation of both YAP1 and EGFR. The activation of EGFR in trametinib‐treated cells, in turn, stimulated hyperactivation of the phosphatidylinositol‐4,5‐bisphosphate 3‐kinase/protein kinase B (PI3K/AKT) pathway as a compensatory survival mechanism. We thus broadened the study to investigate, *in vitro* and *in vivo*, the role of EGFR and PI3K/AKT in limiting trametinib efficacy. Importantly, the findings of this study provide the rationale for using PI3K pathway inhibitors to enhance trametinib efficacy in patients with HNSCC.

## 2 Materials and methods

### 2.1 Bioinformatical analysis of cell lines and TCGA patients

Bulk RNA sequencing (RNA‐seq) and clinical data from HPV16‐negative TCGA‐HNSC patients were obtained from the GDC Data Portal, publicly available HNSCC cell line data was obtained from the Cancer Cell Line Encyclopedia (CCLE) database [20, 21]. Pathway activity scores for 11 relevant oncogenic pathways were calculated using the PROGENy algorithm, supplemental MAPK pathways were downloaded from The Molecular Signatures Database (MSigDB) and their scores were determined using r gsva package [22, 23]. The calcphenotype tool from r oncopredict package was used to infer sensitivity to trametinib using RNA‐seq data and Sanger's Genomics of Drug Sensitivity in Cancer (GDSC) screening data [24]. Statistical analysis was performed using r software.

### 2.2 Cell lines and chemical compounds

The human HNSCC cell lines CAL33 (RRID: CVCL_1108), HSC3 (RRID: CVCL_YA14), HSC4 (RRID: CVCL_1289), FADU (RRID: CVCL_1218), and CAL27 (RRID: CVCL_1107) were purchased from ATCC. The 4NQO‐L cell line was developed in our laboratory by exposing mice to drinking water containing 50 μg·mL^−1^ 4‐nitroquinoline 1‐oxide (4NQO; N8141, Sigma, St. Louis, MO, USA) [25]. All cell lines were maintained at 37 °C in a humidified atmosphere and 5% CO_2_ in DMEM. Cells were routinely tested for mycoplasma infection, and treated with appropriate antibiotics as needed (De‐Plasma™, TOKU‐E, D022). Cells were tested for cell line authentication by the Technion Genomics Center, Technion Israel Institute of Technology, in March 2023. The test was performed using the Promega GenePrint 24 System to determine short tandem repeat (STR) profile of 23 loci plus Amelogenin for gender determination (X or XY). In addition, the male‐specific DYS391 locus is included to identify null Y allele results for Amelogenin. DNA sample from the kit (2800M Control DNA) was included in the analysis and served as positive control for the PCR step. No DNA template was also included as negative control. The results were analyzed using the 3500xl Genetic Analyzer (v.1 data collection software, Life Technologies, Carlsbad, CA, USA) and genemapper idx software (v1.3, Life Technologies). Allelic ladder was included in the run.

### 2.3 Antibodies and reagents

The following antibodies were purchased from Cell Signaling Technology: anti Phospho‐p44/42 MAPK (pErk 1/2) (#4370S), anti p44/42 MAPK (Erk1/2) (#4695) anti pAKT Ser473 (#4060), anti AKT (#4691), anti pS6 S240/244 (#5364), anti‐EGFR (#4267S), anti pEGFR (#2236L), and anti YAP1 (#14074). Anti Ki67 (#275R‐15) was purchased from Cell Marque, and Anti‐actin (691001), from MP Biomedicals, Santa Ana, CA, USA. Erlotinib (HY‐50896) was purchased from MedChemExpress, Monmouth Junction, NJ, USA. Novartis Pharma AG (Basil, Switzerland) provided trametinib (GSK1120212, JTP‐74057), BYL719, and GDC‐0941. All compounds were dissolved in dimethyl sulfoxide (DMSO) for *in vitro* studies and 5% carboxymethylcellulose (CMC) or corn oil with 4% DMSO for *in vivo* experiments.

### 2.4 IC_50_ and synergy assay

Cells were seeded in 96‐well plates (3000–5000 cells per well), treated with increasing concentrations of the drug being tested, and allowed to proliferate for 4 days. At the end point, cells were stained with crystal violet (1 g·L^−1^) for 10 min at room temperature. The crystal violet was then dissolved with 10% acetic acid, and absorbance was measured at 570 nm in a spectrophotometer (Epoch, Biotech, Agilent, Santa Clara, CA, USA). IC_50_ values were calculated using graphpad software (v.7, GraphPad, San Diego, CA, USA). For the synergy assays, the proliferation of the cells in the different treatment groups was determined as the percentage of control (DMSO‐treated) cells; the colorimetric results were analyzed using synergyfinder Software (https://synergyfinder.fimm.fi/).

### 2.5 Phospho‐RTK array

CAL33 and HSC3 cells were seeded in 100‐mm well plates and allowed to adhere overnight. Cells were then treated with 20 nm trametinib or DMSO. Total cell lysates of 300 μg were analyzed using the Proteome Profiler™ Human Phospho‐RTK antibody arrays kit, catalog # ARY001B (R&D Systems, Burlington, ON, Canada), according to the manufacturer's protocol.

### 2.6 Western blotting

As described before [26], Cells were washed with cold phosphate‐buffered saline (PBS) and scraped into lysis buffer supplemented with phosphatase inhibitor cocktail (Stratech, B15001‐BIT, Birkenfeld, Germany). Lysates were cleared by centrifugation at 20 000 ***g*** for 10 min at 4 °C, and supernatants were collected and assayed for protein quantification using Bradford protein assay (Biorad, 5000006). Total lysate, 20 μg, was resolved on NuPAGE 4–12% Bis‐Tris gels and transferred electrophoretically to PVDF membranes (Biorad, 1704159). Membranes were blocked for 1 h in 5% bovine serum albumin (BSA) in Tris‐buffered saline (TBS)‐Tween and then hybridized using the primary antibodies in 5% BSA TBS‐Tween. Mouse and rabbit horseradish peroxidase‐conjugated secondary antibodies (1 : 20 000, GE Healthcare, Chicago, IL, USA) were diluted in 5% BSA in TBS‐Tween. Protein–antibody complexes were detected by chemiluminescence with ECL (Westar Supernova, Cyanogen XLS3.0100, and Westar Nova 2.0 Cyanagen XLS071.0250, Bologna, Italy), and images were captured with a c300 Azure camera system (Dublin, CA, USA). Quantification was done using “image j.” Protein level was calculated relative to loading control (actin) and presented as fold change from the control sample.

### 2.7 Real‐time quantitative PCR

After 24 h of treatment, total RNA was isolated from cells by RNA Mini Kit (Bioline – BIO‐52073, London, UK) according to the manufacturer's protocol. RNA, 1 μg, was converted to cDNA using a qScript cDNA synthesis kit (Quanta Bioscience, 95047‐100) according to the manufacturer's protocol and as previously described [27]. Real‐time PCR was performed (Roche LightCycler® 480 II, Basel, Switzerland) using a prime‐time gene expression master mix (IDT, 1055770) with matching probes: human actin (Hs.PT.39a.22214847 from IDT) or human EGFR (Hs01076078_m1 from ThermoFisher Scientific).

### 2.8 Immunofluorescence

As previously described [25], cells were seeded on glass coverslips in 24‐well plate for 24 h, then rinsed with cold PBS (4 °C) and fixed in 4% PFA for 30 min at room temperature. After that, cells were rinsed with PBS, followed by permeabilization on ice for 5 min in PBS with 0.05% Triton X‐100 (Millipore Sigma, Burlington, MA, USA), re‐rinsed with PBS, and blocked in blocking solution for 30 min at room temperature (5% BSA in Tween‐PBS). The cells were incubated with primary antibodies overnight at 4 °C, rinsed with PBS, and incubated with Cy3‐conjugated anti‐rabbit secondary antibody (1 : 250; 111‐165‐144 Jackson ImmunoResearch, West Grove, PA, USA) at room temperature for 1 h. Cells were then rinsed with PBS and mounted in DAPI Fluoromount‐G (0100‐20, Southern Biotech, Birmingham, AL, USA).

### 2.9 Staining of tumor tissue: immunohistochemistry and immunofluorescence

Immunohistochemistry – As described previously [28], following surgery, tumor tissue samples were obtained from patients with HNSCC at the Ear Nose and Throat Unit of Soroka Medical Center, Be'er Sheva, Israel. The collection of samples was conducted with the patients' understanding and written consent, and the study received approval from the local ethics committee (ethics code: 0421‐16‐SOR and 0103‐17‐SOR) in accordance with the Helsinki Declaration. Tumors were fixed in 4% paraformaldehyde solution overnight at room temperature and then maintained in 70% ethanol until embedding in paraffin. Paraffin‐embedded tumor blocks were sectioned into 5‐μm slices, loaded onto microscope slides, and deparaffinized at 60 °C for 1 h. After additional deparaffinization with a xylene substitute (3803672E, Leica, St. Gallen, Switzerland) and rehydration steps in a descending alcohol series, antigen retrieval was performed. The slides were incubated in 10 mm citric acid buffer, pH 6.0 at 100 °C for 15 min, then cooled in buffer at room temperature and rinsed with doubly distilled water for 3 min, 3′. Thereafter, endogenous peroxidases were inactivated with 0.3% hydrogen peroxide in methanol buffer for 30 min. The slides were washed three times with PBS for 3 min, and then blocked with 5% BSA in PBS‐T (0.1% TWEEN) for 1 h at room temperature. Slides were then incubated overnight at 4 °C with the primary antibodies, Ki67 (Sigma, AB9260, 1 : 200), pMAPK (CST, Danvers, MA, USA, 4370S, 1 : 200), pS6 (CST, 4857S, 1 : 800), and EGFR (CST, 4267S, 1 : 50) diluted in blocking solution. The following day, the slides were washed three times with PBS‐T. The ABC kit (VECTASTAIN®ABC, VE‐PK‐6200) was used for detection according to manufacturer's protocol, with 3,3′‐diaminobenzidine (DAB) (ACH500‐IFU; ScyTek Laboratories, Logan, UT, USA) as a substrate for color development. Slides were counterstained with hematoxylin, dehydrated, and mounted in mounting medium (3801740, Sub‐X, Leica, 3801740). All slides were digitalized using the Panoramic Scanner (3DHISTECH, Budapest, Hungary), and the analysis was performed with qupath‐0.2.3 software [29]. Fields of tumor tissue for analysis were chosen by a blinded investigator. Cells within the analysis field were detected by the qupath‐0.2.3 software and were defined as positive or negative to DAB staining according to a threshold set by two independent blinded investigators (Fig. S1H). Cell detection criteria and thresholds were strictly maintained between compared slides. The specificity of the staining and analysis threshold was verified by comparison with a matched negative control tissue, which was incubated with no primary antibody but subjected to all secondary antibody development processes.

Immunofluorescence – Tissue was processed as described above and then incubated with the primary antibody YAP (CST, 14074, 1 : 500) overnight at 4 °C. The next day, cells were rinsed with PBS‐T and incubated with Alexa Fluor‐647 Anti‐Rabbit secondary antibody (Jackson ImmunoResearch, 111‐605‐144, 1 : 250) at room temperature for 1 h. Finally, cells were washed with PBS‐T and mounted with DAPI Fluoromount‐G® (SouthernBiotech, Birmingham, CA, USA, 0100‐20).

### 2.10 RNA silencing

For the production of shRNA cell lines, we created lentiviruses by transfecting HEK293 cells with the viral plasmids psPAX2, pMD2.G, and PLKO with shRNAs scrambled sequence (designated shControl) or a sequence for the silencing of EGFR expression (designated shEGFR). Transfection was done by using PolyJet transfection reagent (SL100688, SignaGen, Frederick, MD, USA) according to the manufacturer's protocol. Viruses were collected after 48–72 h and used for cell infection. Cells were seeded in 6‐well plates (150 000 cells per well), infected with the lentiviruses in the presence of Polybrene (MilliporeSigma, 5G‐H9268) and selected with puromycin (Thermo Fisher Scientific, A11138‐03).

### 2.11 Cell proliferation assay

Cells were seeded in a 24‐well plate (10 000–20 000 cells per well) and treated the following day. At the endpoint, cells were stained with crystal violet (1 g·L^−1^). Quantification was performed by dissolving out the crystal violet (10% acetic acid) and reading the optical density at 570 nm in a spectrophotometer (Epoch, Biotech).

### 2.12 *In vivo* experiments

Mice were maintained and treated in accordance with the Institutional Animal Care and Use Committee guidelines of Ben‐Gurion University of the Negev. Animal experiments were approved by the Institutional Animal Care and Use Committee (IL‐80‐12‐2015 and IL‐29‐05‐2018). Mice were housed in air‐filtered laminar flow cabinets with a 12‐h light/dark cycle and food and water *ad libitum*. At the end of the experiments, animals were euthanized with CO_2_. To generate CAL33 tumor‐bearing mice, CAL33‐shControl and CAL33‐shEGFR‐3 cells (3 × 10^6^ and 5 × 10^6^ cells, respectively) were suspended in 100 μL of PBS and injected subcutaneously into the right and left flanks of 6‐week‐old female NSG mice (NOD.Cg‐Prkdcscid Il2rgtm1Wjl/SzJ, Jackson Labs, Bar Harbor, ME, USA). Tumor‐bearing mice were then randomized into two groups of five to six mice, based on tumor volume (between 150 and 200 mm^3^). To generate the syngeneic murine model, 4NQL cells (5 × 10^6^ cells) suspended in 50 μL of PBS were injected into the lips of 6‐week‐old female C57BL/6J (WT) mice (Envigo, Huntingdon, UK, C57/BL/6). Tumor‐bearing mice were then randomized into four groups of five to six mice, based on tumor volume (between 150 and 200 mm^3^). To generate HRAS mutant patient‐derived xenografts (PDXs), 3 mm^3^ pieces of tumors were implanted into the right and left flanks of 6‐week‐old female NSG mice (NOD.Cg‐Prkdcscid Il2rgtm1Wjl/SzJ, Jackson Labs). Tumor‐bearing mice were then randomized into two groups of five to six mice, based on tumor volume (between 90 and 120 mm^3^). In all *in vivo* experiments, trametinib (0.5 mg·kg^−1^) was dissolved in corn oil and administered daily by intraperitoneal (i.p.) injection, and BYL719 (25 mg·kg^−1^) was dissolved in 5% CMC and administered daily by oral gavage. Vehicle‐treated mice received both corn oil and 5% CMC. Tumors were measured twice a week with a digital caliper, and tumor volume was calculated according to the formula: *V* = (*L W*′ *W*′)π/2, where *V* is tumor volume, *W* is tumor width, and *L* is tumor length. Tumor volumes are plotted as means ± SEM. In the toxicity study, C57BL/6 female mice were randomized into trametinib/BYL719‐treated and control groups (*n* = 5). Trametinib (0.5 mg·kg^−1^) was dissolved in corn oil and administered by intraperitoneal (i.p.) injection, and BYL719 (25 mg·kg^−1^) was dissolved in 5% CMC and administered daily by oral gavage for seven consecutive days. Vehicle‐treated mice received corn oil and 5% CMC. 24 h after the last treatment, blood was collected from the venous plexus of the eye. Serum was obtained by centrifugation (10 min at 2000 g) for analyte measurements (Biochemical Laboratory at Soroka Medical Center). Hepatic damage was assessed by serum levels of alanine aminotransferase (ALT) and alkaline phosphatase (ALK‐phos). Renal damage was indicated by the levels of blood urea and creatinine. Livers, lungs, kidneys, intestines, or spleens were analyzed for weight and morphology changes.

### 2.13 Statistical analysis

Statistical analysis was performed using graphpad prism software versions 7 and 9, and results are presented as means ± SEM. For comparisons between two groups, *P* values were calculated using the unpaired *t*‐test (*P < 0.05, **P < 0.01, ***P < 0.001). For comparisons between three or more groups, *P* values were calculated using one‐way ANOVA.

## 3 Results

### 3.1 Trametinib treatment leads to YAP1 activation and EGFR upregulation

**Fig. 1:**
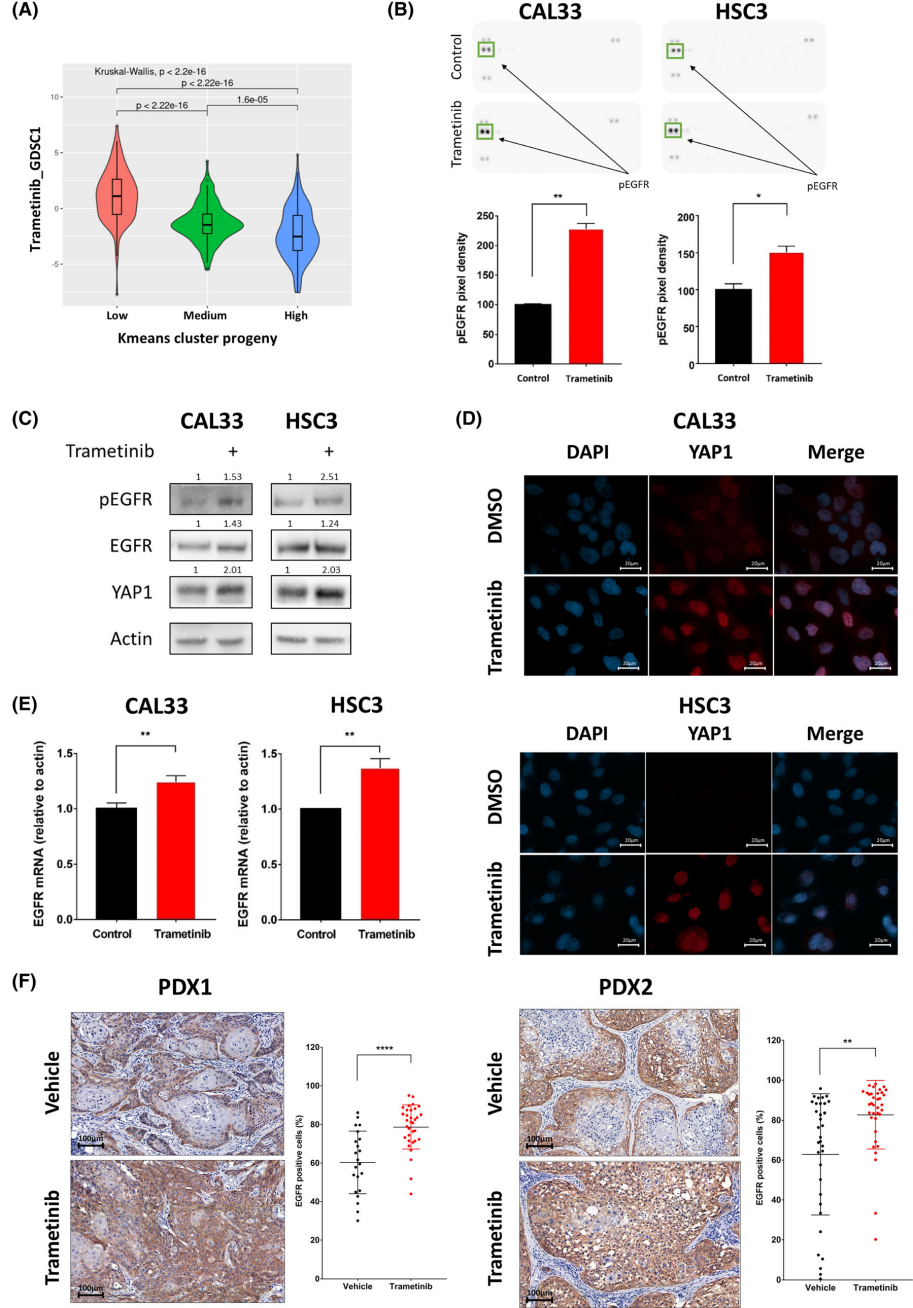
Trametinib treatment leads to yes‐associated protein 1 (YAP1) and epidermal growth factor receptor (EGFR) expression and activation. (A) Violin plot represents the predicted trametinib sensitivity score calculated using Sanger's Genomics of Drug Sensitivity in Cancer 1 (GDSC1) screening data in previously established mitogen‐activated protein kinase (MAPK)‐activity groups. (B) (top) Protein expression (PathScan® protein array) of CAL33 and HSC3 cells treated with 20 nm trametinib for 24 h; (bottom) quantified phosphorylated‐EGFR expression level in protein array. Error bars indicate SD. Data from single experiment with two technical repeats. (C) Western blot for the indicated proteins following 24 h of treatment with 20 nm trametinib. Numbers indicate the fold change in protein level normalized to Actin. Data represent a representative experiment from two independent experiments. (D) Representative pictures (*n* = 10, from two independent experiments) of immunofluorescence staining for YAP1 expression and localization. Nuclei are visualized by DAPI staining (X100, 20 μm). (E) mRNA levels of EGFR following 24 h of 20 nm trametinib treatment in CAL33 and HSC3 head and neck squamous cell carcinoma cell lines. Error bars indicate SD. Data represent a representative experiment from three independent experiments. (F) Representative immunohistochemical staining of EGFR in tissue samples (X20, 100 μm) and quantification of EGFR cells (%, per field) after 25 days of treatment with vehicle (PDX1 *n* = 4 tumors and *n* = 22 analysis fields; PDX2 *n* = 4, and *n* = 33 analysis fields) or trametinib (0.5 mg·kg^−1^) (PDX1 *n* = 3 tumors and *n* = 33 analysis fields; PDX2 *n* = 4, and *n* = 36 analysis fields). Error bars indicate SD. Statistical significance was calculated using the unpaired *t*‐test (*P < 0.05, **P < 0.01, ****P < 0.0001).

To explore if activation of the MAPK pathway is associated with sensitivity to trametinib in HNSCC patients, we used the gene expression profiling of HPV‐negative HNSCC patients from the TCGA to assess pathways activation using the PROGENy algorithm. The activity scores of 11 highly relevant oncogenic pathways were used to perform k‐means clustering of patients. We identified three clusters with low, moderate, or high MAPK pathway activity (Fig. S1A), and verified that the MAPK pathway activity is significantly different between the three groups (Fig. S1B). Next, the calcPhenotype tool from r oncopredict package was used to infer the sensitivity to trametinib using RNA‐seq data and Sanger's Genomics of Drug Sensitivity in Cancer (GDSC) screening data [24]. Samples with low MAPK activity had significantly lower predicted sensitivity to trametinib than samples with high MAPK activity (Fig. 1A and Fig. S1C), which points to MAPK‐activated patients being potentially better responders to trametinib.

To evaluate the sensitivity of HNSCC cell lines to trametinib, we first measured the half‐maximal inhibitory concentration (IC_50_) of trametinib in five human cell lines (HSC3, CAL33, HSC4, FADU, and CAL27). All five cell lines showed susceptibility to trametinib, with IC_50_ values ranging between 40 and 281 nm (Fig. S1D), and exhibited MAPK pathway activation based on gene signature (Fig. S1E). Because upregulation and activation of RTKs is a common response to treatment with targeted therapies [28], including trametinib [30], we characterized the activation status of key RTKs in CAL33 and HSC3 HNSCC cells treated with DMSO or trametinib by using phospho‐RTK arrays. Among the 42 RTKs tested, phosphorylated‐EGFR was the most abundant receptor and was significantly upregulated following trametinib treatment in both cell lines (Fig. 1B, Fig. S1F). Because previous studies had shown that the transcription co‐regulator YAP1 is upregulated following trametinib treatment [19] and that YAP1 can mediate EGFR overexpression [31], we hypothesized that trametinib treatment would induce both YAP1 and EGFR overexpression and activation in treated HNSCC cell lines. To test this premise, we measured YAP1 and EGFR expression and activation using western blot, and we examined the localization of YAP1 using immunofluorescence. We observed an increase in YAP1 protein level following trametinib treatment, along with EGFR upregulation and activation (Fig. 1C). Immunofluorescence staining confirmed increased YAP1 expression and demonstrated its nuclear accumulation after trametinib treatment (Fig. 1D). The increase in YAP1 expression was associated with a significant increase in the level of EGFR mRNA (Fig. 1E).

To verify YAP1 translocation and the upregulation of EGFR expression in preclinical models, we stained YAP1 and EGFR in tumor tissues of two patient‐derived xenografts (PDX), namely, PDX1 and PDX2, that were treated with trametinib in NSG mice and showed tumor growth delay [27]. Immunofluorescent staining showed translocation of YAP1 after trametinib treatment in PDX2 (Fig. S1G). Immunohistochemical analysis comparing the EGFR expression between trametinib‐treated and vehicle‐treated PDXs showed that trametinib treatment induced EGFR expression in most tumor cells. In contrast, in the vehicle‐treated mice, many tumor cells showed low or no expression of EGFR (Fig. 1F). These results suggest that EGFR overexpression and activation is an immediate adaptive response to trametinib that may limit trametinib efficacy.

### 3.2 EGFR limits trametinib efficacy by hyperactivating the PI3K/AKT pathway

**Fig. 2:**
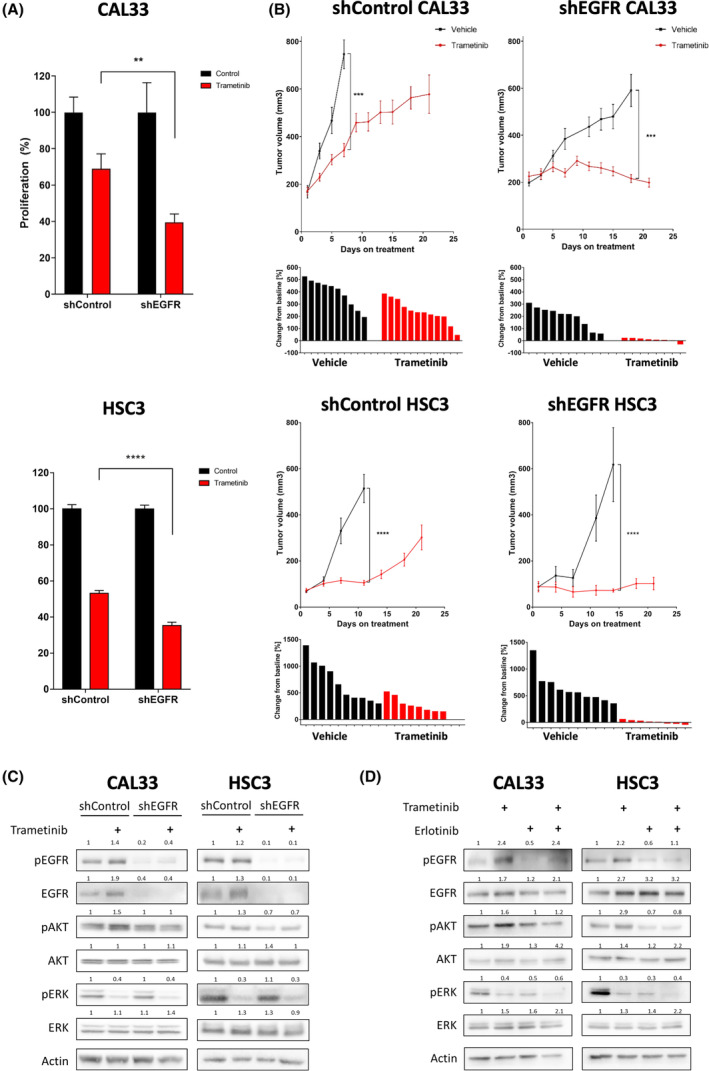
Epidermal growth factor receptor (EGFR) limits trametinib efficacy by hyperactivating the phosphatidylinositol‐4,5‐bisphosphate 3‐kinase/protein kinase B (PI3K/AKT) pathway. (A) 4‐day proliferation assay testing trametinib (50 nm) efficacy in shControl and shEGFR CAL33 and HSC3 cell lines. Error bars indicate SEM. Data represent a representative experiment from three independent experiments. (B) (top) Tumor volume of the shControl or shEGFR CAL33 and HSC3 cell‐derived xenografts injected to NSG mice subsequently treated daily with trametinib (0.5 mg·kg^−1^ i.p.) or vehicle (*n* = 5). Error bars indicate SEM. (bottom) change in tumor volume from first to last day of treatment. (C) Western blot for the indicated proteins following 24 h of 20 nm trametinib treatment in shControl and shEGFR‐3 CAL33 and HSC3 cell lines. Numbers indicate the fold change in protein level normalized to Actin. Data represent a representative experiment from three independent experiments. (D) Western blot for the indicated proteins following 24 h of 20 nm trametinib treatment with or without 5 μm EGFR inhibitor erlotinib, in CAL33 and HSC3 head and neck squamous cell carcinoma cell lines. Numbers indicate the fold change in protein level normalized to Actin. Data represent a representative experiment from three independent experiments. Statistical significance was calculated using the unpaired *t*‐test (**P < 0.01, ***P < 0.001, ****P < 0.0001).

To investigate the potential role of EGFR in limiting trametinib efficacy, we reduced the expression of EGFR by shRNA in the CAL33 and HSC3 cell lines (Fig. S2A) and then evaluated the sensitivity of tumor cells to trametinib *in vitro* and *in vivo*. Proliferation assay showed that EGFR knockdown sensitized both cell lines to trametinib (Fig. 2A). To test the effect of EGFR expression on the efficacy of trametinib in mice, we injected shEGFR and shControl cells subcutaneously into NSG mice, and when tumors reached ~ 200 mm^3^ in size, treatment with trametinib was initiated. Tumor growth kinetics showed that following exposure to trametinib shControl tumors exhibited a growth delay, but all tumors eventually progressed. In contrast, trametinib treatment of shEGFR tumor‐bearing mice resulted in stable disease, and tumor progression was not detected even after 22 days of treatment (Fig. 2B).

To obtain further insights into the downstream signaling of EGFR that may be responsible for limiting the efficacy of trametinib, we examined the phosphorylation status of AKT by western blot analysis of both shControl and shEGFR cell lines treated with trametinib or DMSO. In shControl cells, trametinib treatment induced EGFR overexpression and activation along with hyperactivation of the PI3K/AKT pathway, as indicated by an increase in pAKT. In contrast, in shEGFR cells, trametinib treatment did not induce pAKT upregulation (Fig. 2C). We confirmed the role of EGFR in activating the PI3K/AKT pathway by pharmaceutically inhibiting EGFR signaling with erlotinib. In that experiment, we evaluated the expression of EGFR, PI3K/AKT, and MAPK signaling in CAL33 and HSC3 cell lines treated with trametinib, erlotinib, or a combination of erlotinib and trametinib. EGFR blockage prevented trametinib‐induced PI3K/AKT hyperactivation in both CAL33 and HSC3 cell lines (Fig. 2D). Overall, these *in vivo* and *in vitro* results indicate that EGFR plays a role in limiting the efficacy of trametinib, putatively via activation of the PI3K/AKT pathway.

### 3.3 Preventing PI3K/AKT hyperactivation enhances trametinib efficacy

**Fig. 3:**
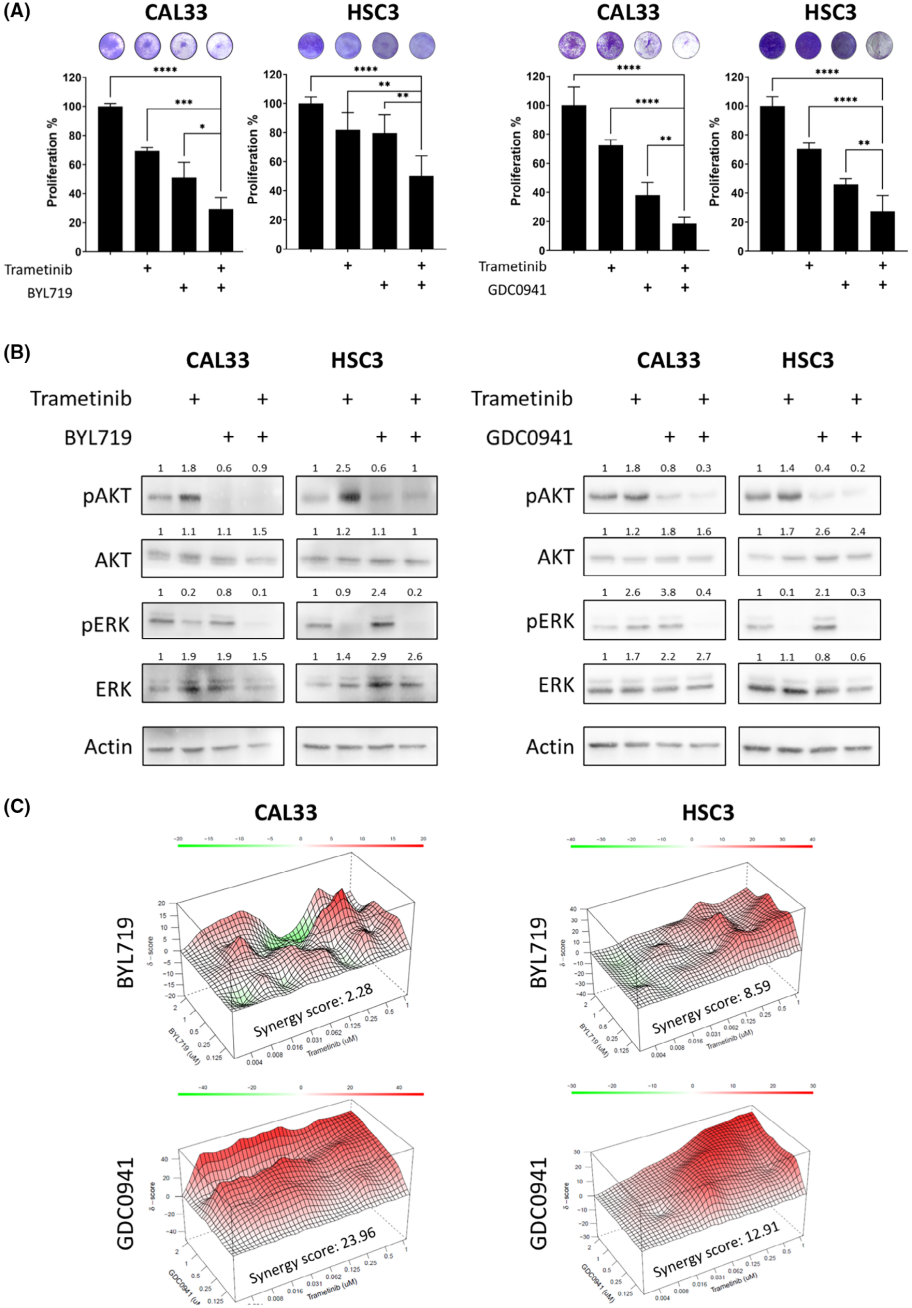
Dual treatment with trametinib and a phosphatidylinositol‐4,5‐bisphosphate 3‐kinase (PI3K) inhibitor has a synergistic effect *in vitro*. (A) Four‐day proliferation assay testing the efficacy of trametinib (20 nm) and of BYL719 (2 μm; the isoform‐specific inhibitor of PI3K), or GDC‐0941 (1 μm; the inhibitor of α and δ PI3K), alone or in combination, in CAL33 and HSC3 head and neck squamous cell carcinoma (HNSCC) cell lines. Error bars indicate SD. Data represent a representative experiment from three independent experiments. (B) Western blot for the indicated proteins following 24 h of 20 nm trametinib treatment with or without BYL719 (2 μm) or GDC‐0941 (1 μm) in CAL33 and HSC3 HNSCC cell lines. Continued use of tumor cell lysates from Fig. 1B. Numbers indicate the fold change in protein level normalized to Actin. Data represent a representative experiment from three independent experiments. (C) Synergy scores and heatmap calculated by SynergyFinder for the combination of trametinib with BYL719 or GDC‐0941 in CAL33 and HSC3 HNSCC cell lines. Data represent a representative experiment from three independent experiments. Statistical significance was calculated using one‐way ANOVA (*P < 0.05, **P < 0.01, ***P < 0.001, ****P < 0.0001).

To explore whether hyperactivation of the PI3K/AKT pathway following trametinib treatment is a robust phenomenon, we determined pAKT in response to trametinib in three additional HNSCC cell lines (HSC4, FADU, and CAL27). Western blot analysis showed that four of the five cell lines exhibited an increase in pAKT within 24 h of trametinib treatment (Fig. S2B). Therefore, we hypothesized that blockage of PI3K/AKT pathway with a PI3K inhibitor would enhance trametinib efficacy. To validate this premise, we investigated the biological and molecular response of CAL33 and HSC3 cells to treatment with trametinib with and without BYL719 (alpelisib), a p110a isoform‐specific inhibitor of PI3K, or GDC‐0941 (pictilisib), a pan PI3K inhibitor. A four‐day proliferation assay showed that combining trametinib with BYL719 or GDC‐0941 limited the proliferation of cells vs. single‐drug treatment (Fig. 3A, Fig. S3A). Protein expression analysis showed that a combined treatment with trametinib and either BYL719 or GDC‐0941 prevented the trametinib‐induced activation of the PI3K/AKT pathway in both tested cell lines (Fig. 3B).

To explore whether the antiproliferative effect of trametinib in combination with BYL719 or GDC‐0941 is additive or synergistic, a synergy test was performed in HSC3 and CAL33 HNSCC cell lines. In this test, an average calculated synergy score above 1 indicates a synergistic antitumor effect between trametinib and the PI3K inhibitor. The synergy score of GDC‐0941/trametinib was higher than that for BYL719/trametinib in both models (Fig. 3C). To corroborate the findings, we performed similar experiments in other HNSCC cell line models, namely, HSC4, FADU, and CAL27. We found that BYL719/trametinib has a synergistic antitumor effect in CAL27, while GDC‐0941/trametinib has synergistic effects in HSC4 and CAL27 (Fig. S3B). Overall, we found that in most HNSCC models tested, trametinib treatment induced hyperactivation of the PI3K/AKT pathway and that blocking PI3K signaling has a synergistic antitumor effect when combined with trametinib.

### 3.4 Dual treatment with trametinib and BYL719 prolongs PDX growth arrest in mice

**Fig. 4:**
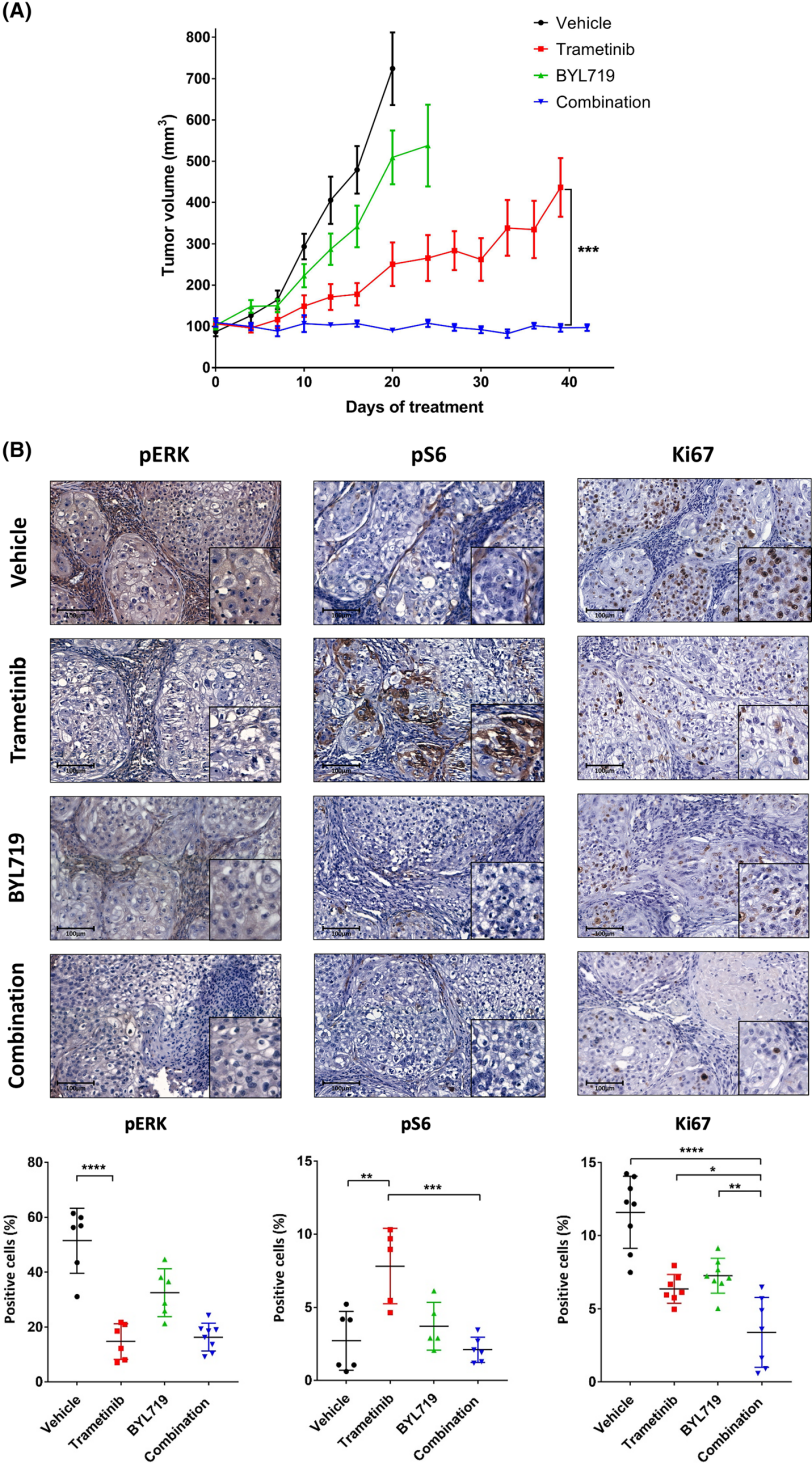
Dual treatment with trametinib and BYL719 prolongs patient‐derived xenograft (PDX) growth arrest in mice. (A) Tumor volume of the HRAS‐mutated PDX3 model transplanted in NSG mice. Mice were randomized into four arms (*n* = 5) and treated daily with trametinib (0.5 mg·kg^−1^ i.p.), BYL719 (25 mg·kg^−1^ by oral gavage), a combination BYL719/trametinib treatment or vehicle. Error bars indicate SEM. (B) Representative immunohistochemical staining (X20, 100 μm) and the quantification of cells positive for: phosphorylated mitogen‐activated protein kinase (pERK) staining (left) indicating the response to trametinib treatment (*n* = 4 tumors and *n* = 6 analysis fields), pS6 staining (middle) indicating activation of the phosphatidylinositol‐4,5‐bisphosphate 3‐kinase/protein kinase B/mammalian target of rapamycin (PI3K/AKT/mTOR) pathway (*n* = 4 tumors and *n* = 6 analysis fields), and Ki67 staining (right) indicating cell proliferation (*n* = 4 tumors and *n* = 8 analysis fields), after 4 days of treatment. Error bars indicate SD. Statistical significance was calculated using one‐way ANOVA (*P < 0.05, **P < 0.01, ***P < 0.001, ****P < 0.0001).

Since BYL719 was recently approved by the FDA [32] for the treatment of certain breast cancers and is in clinical development for HNSCC (NCT04997902), after promising results in a phase Ia clinical trial [33], we chose this agent for further investigation. To test the antitumor activity of trametinib, BYL719, and their combination *in vivo*, we used an HRAS‐mutated PDX model (designated here PDX3). Specifically, we implanted PDX3 into NSG mice, and after successful engraftment mice were randomized into four arms. The mice were treated daily with vehicle, trametinib (0.5 mg·kg^−1^), BYL719 (25 mg·kg^−1^), or a combination of both agents. Tumor growth kinetics showed that PDX3 was resistant to BYL719 treatment but responsive to trametinib monotherapy, as indicated by the delay in tumor progression. However, marked tumor growth arrest was observed in mice treated with the BYL719/trametinib combination, with no detectable increase in tumor volume, even after 40 days of treatment (Fig. 4A, Fig. S4A). Importantly, the combination treatment with the two agents did not result in any observed toxicity, as the animals' weight remained stable during the efficacy experiment. Further biochemical tests of serum markers after 7 days of treatment showed no major liver or kidney toxicity besides a reduction in the creatinine levels in mice treated with BYL719 and trametinib (Fig. S4C–E).

To quantify the on‐target effect of the treatments on the activity of signaling pathways in tumor cells and on tumor cell proliferation, we used immunohistochemical staining to examine tumors after 4 days of treatment. For evaluating activation of the MAPK and AKT pathways, we stained the tumors for pERK and pS6 as the downstream activator of AKT and mammalian target of the rapamycin (mTOR) pathway, respectively. As expected, tumors treated with trametinib monotherapy showed a reduction of pERK staining but simultaneously an increase in pS6 staining (an indicator for AKT/mTOR pathway activation) and EGFR expression (Fig. 4B, and Fig. S4B). Staining with the proliferation marker of Ki67 showed decreased tumor cell proliferation in tumors treated with either trametinib or BYL719 monotherapy. However, more significant inhibition of cell proliferation was detected in tumors treated with the BYL719/trametinib combination (Fig. 4B). These results indicate that the dual treatment inhibited the PI3K/AKT/mTOR and MAPK pathways, both which are needed for tumor cell proliferation.

### 3.5 Treatment with trametinib and BYL719 in a syngeneic murine model leads to tumor shrinkage

**Fig. 5:**
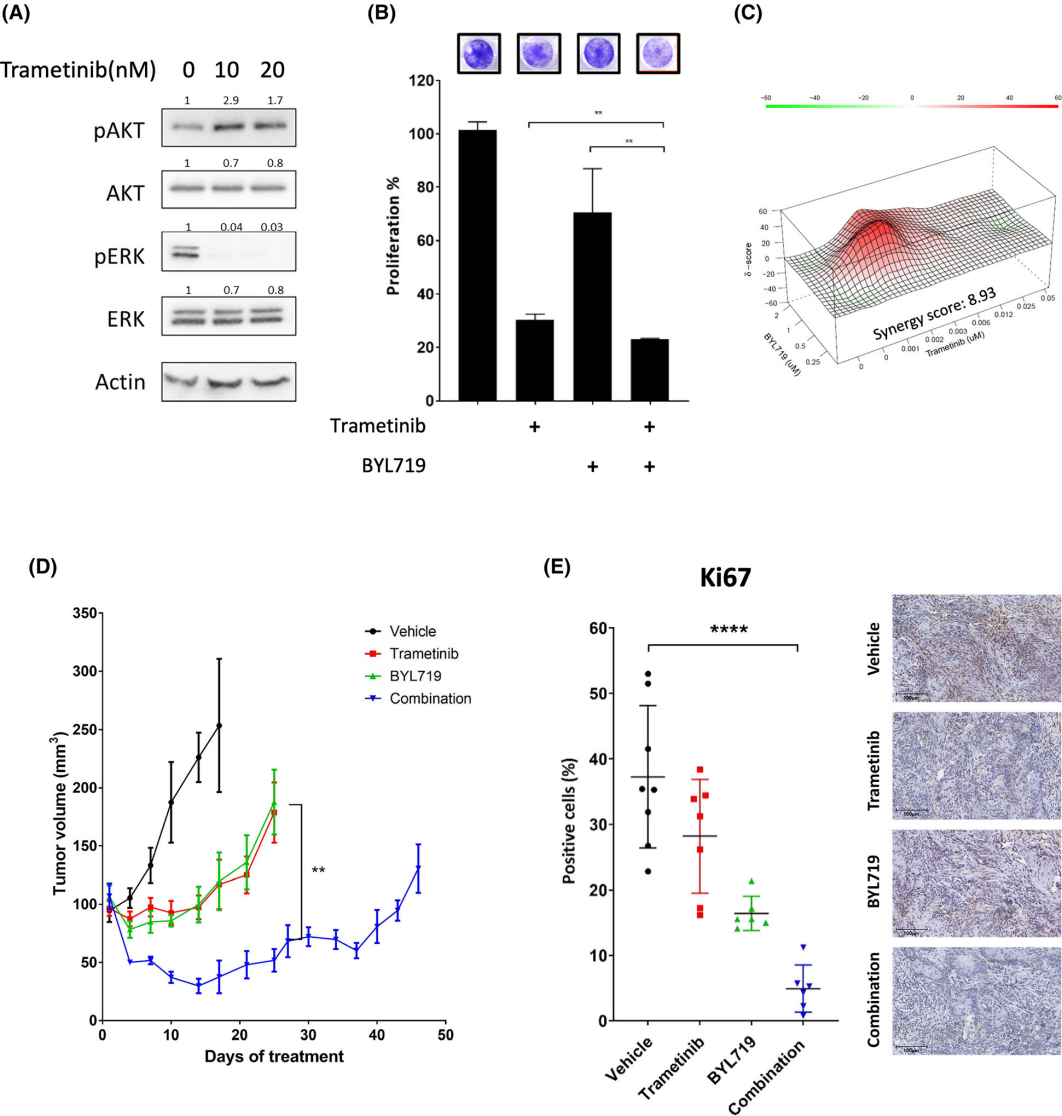
Treatment with trametinib and BYL719 in a syngeneic murine model leads to tumor shrinkage. (A) Western blot for the indicated proteins following 24 h of treatment with the indicated concentrations of trametinib in the 4NQO‐L murine head and neck cancer (HNC) cell line. Numbers indicate the fold change in protein level normalized to Actin. Data represent a representative experiment from three independent experiments. (B) Four‐day proliferation assay testing the efficacy of trametinib (20 nm), with and without BYL719 (2 μm) in the murine 4NQO‐L HNC cell line. Error bars indicate SD. Data represent a representative experiment from three independent experiments. (C) Synergy scores and heatmap calculated by SynergyFinder for the combination of trametinib and BYL719 in the 4NQO‐L murine HNC cell line. Data represent a representative experiment from three independent experiments. (D) Tumor volume of the 4NQO‐L xenograft model in C57BL/6J (WT) mice; 3 × 10^6^ cells were injected subcutaneously, mice were randomized into four arms (*n* = 6) treated daily with trametinib (0.5 mg·kg^−1^ i.p.), BYL719 (25 mg·kg^−1^ by oral gavage), a combination treatment, or vehicle. Error bars indicate SEM. (E) Representative images of Ki67 immunostained tissue sections (X20, 100 μm) and quantification of positive cells (*n* = 4 tumors and *n* = 8 analysis fields). Error bars indicate SD. Statistical significance was calculated using one‐way ANOVA (**P < 0.01, ****P < 0.0001).

To corroborate our findings in a physiologically relevant system, we used a KRAS‐mutant cell line derived from the lip of 4NQO‐induced carcinogenesis, known as 4NQO‐L cell line [25]. In response to exposure to trametinib, 4NQO‐L cells exhibited a rapid upregulation of pAKT concomitant with a down‐regulation of pERK (Fig. 5A). Blocking the PI3K/AKT pathway with BYL719 enhanced trametinib efficacy, resulting in a significant reduction in cell number (Fig. 5B). The synergy score of 8.93 for the BYL719/trametinib combination in 4NQO‐L cells was high relative to that for the human cell lines (Fig. 5C, Fig. S5A).

The promising *in vitro* results provided the grounds for us to explore the efficacy of trametinib and BYL719 therapies, alone and in combination, in WT mice injected orthotopically with 4NQO‐L cells. Treatment of 4NQO‐L‐tumor‐bearing mice with trametinib or BYL719 monotherapies awarded a temporary delay in tumor growth for 15 days, but detectable progression was measured after 20 days of treatment. In contrast, the combination BYL719/trametinib treatment resulted in rapid tumor shrinkage, which lasted ~ 30 days (Fig. 5D), with no evidence of toxicity (Fig. S5B). Histopathological staining of the proliferative marker Ki67 shows a decreased number of stained cells in tumors treated with either trametinib or BYL719 monotherapy compared with the vehicle‐treated group. However, combining the two treatments substantially reduced tumor cell proliferation (Fig. 5E). Overall, these results provide confirmation of AKT pathway hyperactivation as a robust and physiologically relevant cellular response to trametinib treatment and emphasize the potential of PI3K inhibitors in enhancing trametinib efficacy.

## 4 Discussion

In this study, we found that the efficacy of treatment with the MEK1/2 inhibitor, trametinib, is impaired by rapid hyperactivation and upregulation of YAP1 and EGFR. YAP1 overexpression and its localization in the nucleus is associated with the upregulation of EGFR transcription in trametinib‐treated cells. In trametinib‐treated cells, overactivation of EGFR induces PI3K/AKT pathway signaling, which limits treatment efficacy *in vitro* and *in vivo*. Thus, blocking the PI3K/AKT pathway with BYL719 enhanced trametinib efficacy, and the BYL719/trametinib combination showed superior antitumor activity in HNSCC cell lines, PDXs, and in a syngeneic murine HNSCC model.

Although mutations in key MAPK‐pathway genes are infrequent in HNC patients, many HNC tumors rely on the MAPK pathway for proliferation and survival [9]. Several reports have shown that MAPK inhibition is potent in HNSCC models [11, 19, 34, 35, 36, 37, 38]. Indications of the clinical potential of blocking the MAPK pathway were evident in a window‐of‐opportunity trial showing that trametinib induced tumor shrinkage in 65% of patients with HNSCC [18]. Our data further support this tumor cell dependency on the MAPK pathway, as all tested HNSCC cell lines responded to trametinib in nanomolar concentrations. The knowledge that resistance develops over time [19] led us to investigate the intracellular signaling adaptations that allow tumor cells to escape from this treatment. We observed that EGFR overexpression and hyperactivation of the PI3K/AKT pathway is a common mechanism of response to trametinib in HNSCC cell lines, PDXs, and a syngeneic HNSCC model. The upregulation and activation of EGFR in response to MAPK pathway inhibition have been described in several cancer types and are known to limit the effect of MEK inhibitors [39, 40, 41, 42], but to date and to the best of our knowledge, upregulation of EGFR in response to inhibition of the MAPK pathway has not been studied in HNSCC. Thus, our observation of EGFR overexpression and activation in response to trametinib treatment provides a novel molecular resistance mechanism to trametinib therapy in HNSCC. Untreated tumors showed EGFR expression in tumor cells within the “ecological transition zone,” where tumor and host cells interact [43]. However, after treatment with trametinib, EGFR expression in tumor cells became independent of the proximity between tumor cells and host cells.

EGFR expression is regulated by multiple means, including transcriptionally by specificity protein 1 (SP1), eukaryotic translation termination factor 1 (ETF1), T‐cell factor (TCF) [44], and TEAD [31]. The activity of TEAD is known to be mediated by the transcription co‐regulator YAP1 [45, 46, 47]. Mudianto et al. [19] reported YAP1‐mediated resistance to trametinib in HNSCC cell lines and described AKT maintained pathway activation in some trametinib‐acquired resistant models. Our results align with YAP1 upregulation and AKT pathway activation and suggest the mechanism by which trametinib induces the AKT pathway activation via EGFR. To the best of our knowledge, there is only one study with contradictory results, that is, showing that a high dosage of trametinib reduced EGFR expression in three HNC cell lines through its effect on c‐MYC expression [34]. The differences between that study and the current study may derive from differences in cell lines, times of analysis, and concentrations of trametinib. While MAPK pathway activation was shown to be involved in YAP1 translocation and activation [19, 48, 49, 50, 51] in human malignancies, additional research is required to determine the exact mechanisms involved in YAP1 translocation, and its effect on the transcriptional regulation of EGFR expression in response to trametinib treatment in HNSCC cell lines.

EGFR regulates several signaling pathways, including the RAS–RAF–MEK–ERK and the PI3K‐AKT–mTOR axes, as well as SRC‐like family kinases, PLCγ‐PKC, and STATs [52]. Activation of such parallel oncogenic pathways bypasses the inhibitory effect of trametinib and other MEK inhibitors [53]. In particular, the PI3K pathway stands out as a major resistance mechanism to MEK inhibition [54], which aligns with our findings. Multiple mechanisms for MAPK feedback regulation of AKT signaling have been described, including modulation of GAB and IRS scaffolding proteins that interact with protein from both pathways [55], and feedback regulation of RTK expressions, such as HER2, HER3, and IGF1R [56, 57, 58]. Our data show that the PI3K/AKT hyperactivation is dependent on EGFR expression and activation, as EGFR knockout or pharmacological inhibition of EGFR is sufficient to block trametinib‐induced feedback activation of PI3K/AKT pathway and improve the duration of response to trametinib.

Combination treatments with MEK and EGFR inhibitors have been previously investigated in preclinical and clinical settings, especially in the context of resistance to anti‐EGFR monotherapy and of BRAF or RAS mutated tumors [59, 60, 61, 62, 63, 64]. Specifically, the combination of a MEK inhibitor with afatinib, a pan HER family inhibitor was shown to have a synergistic effect in HNSCC [64, 65]. Our results on the key role EGFR plays in bypassing the inhibitory effect of trametinib suggest that the combination of EGFR inhibitors with MEK inhibitors may be beneficial not only in the context of improving the efficacy of EGFR inhibitors but also when treating with MEK inhibitors.

As demonstrated previously in several cancer types, dual treatment blocking both the PI3K/AKT and MAPK pathways showed a synergistic effect [66]. Of the two PI3K inhibitors that we tested, the combination of trametinib with GDC‐0941 seemed to have a stronger synergistic effect across cell lines than the combination with BYL719. This finding may be explained by the fact that GDC‐0941 inhibits the p110 α and δ subunits of PI3K, while BYL719 is an α‐specific inhibitor [67]. Despite the superior activity of the combination with GDC‐0941 over BYL719 in culture, we chose to proceed with our investigation on therapy combination *in vivo* with BYL719 due to its promising results in clinical trials [33] and the reduced risk of side effects due to its specificity.

## 5 Conclusions

This study revealed that EGFR overexpression and activation are responsible for limiting trametinib efficacy by activating the PI3K/AKT pathway and that blockage of the EGFR or PI3K is crucial for prolonging the response to trametinib treatment.

## Conflict of interest

LGTM is listed as an inventor on intellectual property owned by Memorial Sloan Kettering Cancer Center and licensed to PGDx, unrelated to this work. The other authors declare no conflict of interest.

## Author contributions

ABS, ON, and ME designed the experiments. ABS, ON, DM, SJ, JZ, MP, TBL, RAS, KMY, YL, CB, and DK performed the experiments. LGTM, FK, CC‐L, IK, and JH preformed the bioinformatic analysis. ON and ME wrote the manuscript with input from SJ, LGTM, IK, JH, and DK.

### Peer review

The peer review history for this article is available at https://www.webofscience.com/api/gateway/wos/peer‐review/10.1002/1878‐0261.13500.

## Supporting information


**Fig. S1.** Sensitivity to trametinib in HNSCC. (A) Heatmap represents the 11 PROGENy pathway scores for primary tumor samples from HPV16‐Negative TCGA‐HNSSC patients, the scores are clustered according to k‐means clustering (k = 3) and clusters are named according to the MAPK activity. (B) Violin plot represents PROGENy MAPK score for the different clusters. (C) Violin plot represents predicted Trametinib sensitivity score calculated using Sanger's Genomics of Drug Sensitivity in Cancer 2 (GDSC2) screening data in previously established MAPK‐activity groups. (D) IC_50_ values indicating sensitivity to trametinib after 96 h of treatment. Data represent a representative experiment from three independent experiments. Error bars indicate SD. (E) Heatmap shows unsupervised hierarchical clustering of CCLE HNSCC cell lines based on GSVA scores for selected MAPK gene signatures and MAPK PROGENy pathways. (F) High and low exposure of protein array (PathScan® protein array) blots of CAL33 and HSC3 cells treated with 20 nM trametinib for 24 h. (G) Representative immunofluorescent staining of YAP1 (red) and DAPI (blue) in tissue samples (X20, 50 μm) after 25 days of treatment with vehicle or trametinib (0.5 mg/kg). (H) Example of the analysis method and detection threshold for EGFR quantification in QuPath software. Data represent a representative experiment from two independent experiments.

**Fig. S2.** AKT activation by trametinib treatment. (A) Western blot for Epidermal growth factor receptor (EGFR) protein levels in CAL33 and HSC3 cells infected with control or shEGFR‐1, shEGFR‐2, shEGFR‐3 or shEGFR‐4 PLKO silencing vectors. (B) Western blot for the indicated proteins following 2 and 24 h of treatment with 20 nM trametinib in five human cell lines (CAL33, HSC3, HSC4, FADU, and CAL27). Numbers indicate the fold change in protein level normalized to actin. Data represent a representative experiment from two independent experiments.

**Fig. S3.** Efficacy of dual treatment in HNSCC cell lines. (A) 4 Four‐day proliferation assay testing the efficacy of trametinib (20 nM) and of BYL719 (2 μM), or GDC‐0941 (1 μM), alone or in combination, in HSC4, CAL27, and FADU HNSCC cell lines. Data represent a representative experiment from three independent experiments. Error bars indicate SD. Statistical significance was calculated using one‐way ANOVA (ns – not significant, *p < 0.05, **p < 0.01, ***p < 0.001, ****p < 0.0001). (B) Synergy scores and heat map calculated by SynergyFinder for the combination of trametinib with BYL719 or GDC‐0941 in HSC4, CAL27, and FADU HNSCC cell lines. Data represent a representative experiment from three independent experiments.

**Fig. S4.** Efficacy and toxicity of combination therapy. (A) Image of the dissected tumors at end point of the experiment. (B) Representative immunohistochemical staining of epidermal growth factor receptor (EGFR) in tissue samples of PDX‐3 (X20, 100 μm) and quantification of EGFR cells (%, per field) after 20–25 days of treatment with vehicle or trametinib (0.5 mg/kg) (*n* = 4 tumors and *n* = 50 analysis fields). Error bars indicate SD. Statistical significance was calculated using the unpaired t‐test (****p < 0.0001). (C) Monitoring of body weight of PDX3‐implanted NSG mice treated with vehicle, trametinib (0.5 mg/kg) by intraperitoneal injection, BYL719 (25 mg/kg) by oral gavage, or a BYL719/trametinib combination treatment for 40 days. Error bars indicate SD. (D) Organ weight of C57BL/6J (WT) mice (*n* = 5) treated for 7 days with vehicle (corn oil, i.p, and 5% CMC, oral gavage) or trametinib (0.5 mg/kg, i.p) and BYL719 (25 mg/kg, oral gavage). Line indicated median. (E) serum levels of alanine aminotransferase (ALT) and alkaline phosphatase (ALK‐phos), urea and creatinine after 7 days of treatment with vehicle (corn oil, i.p, and 5% CMC, oral gavage) or trametinib (0.5 mg/kg, i.p) and BYL719 (25 mg/kg, oral gavage). Line indicated median.

**Fig. S5.** Efficacy and toxicity in syngeneic HNC murine model. (A) Synergy scores and heatmap calculated by SynergyFinder for the combination of trametinib with GDC‐0941 in a 4NQO‐L murine HNSCC cell line. Data represent a representative experiment from three independent experiments. (B) Body weight of C57BL/6J (WT) mice bearing 4NQO‐L tumors treated with vehicle, trametinib (0.5 mg/kg) by intraperitoneal injection, BYL719 (25 mg/kg) by oral gavage, or with a BYL719/ trametinib combination treatment. Error bars indicate SD.

## Data Availability

The data that support the findings of this study are available in Figs 1, 2, 3, 4, 5 and/or the supplementary material of this article. Data used in the bioinformatic analysis of cell lines and patients are publicly available as indicated in the materials and methods section. The source code used for the bioinformatic analysis is available in GitHub (https://github.com/DKFZ‐E220/Trametinib).
