# Supplementary figures and images for: Activation of the EGFR/PI3K/AKT pathway limits the efficacy of trametinib treatment in head and neck cancer
Source: Mol Oncol. 2023 Aug 31;17(12):2618–36. doi: 10.1002/1878-0261.13500 (PMC10701778; doi:10.1002/1878-0261.13500)

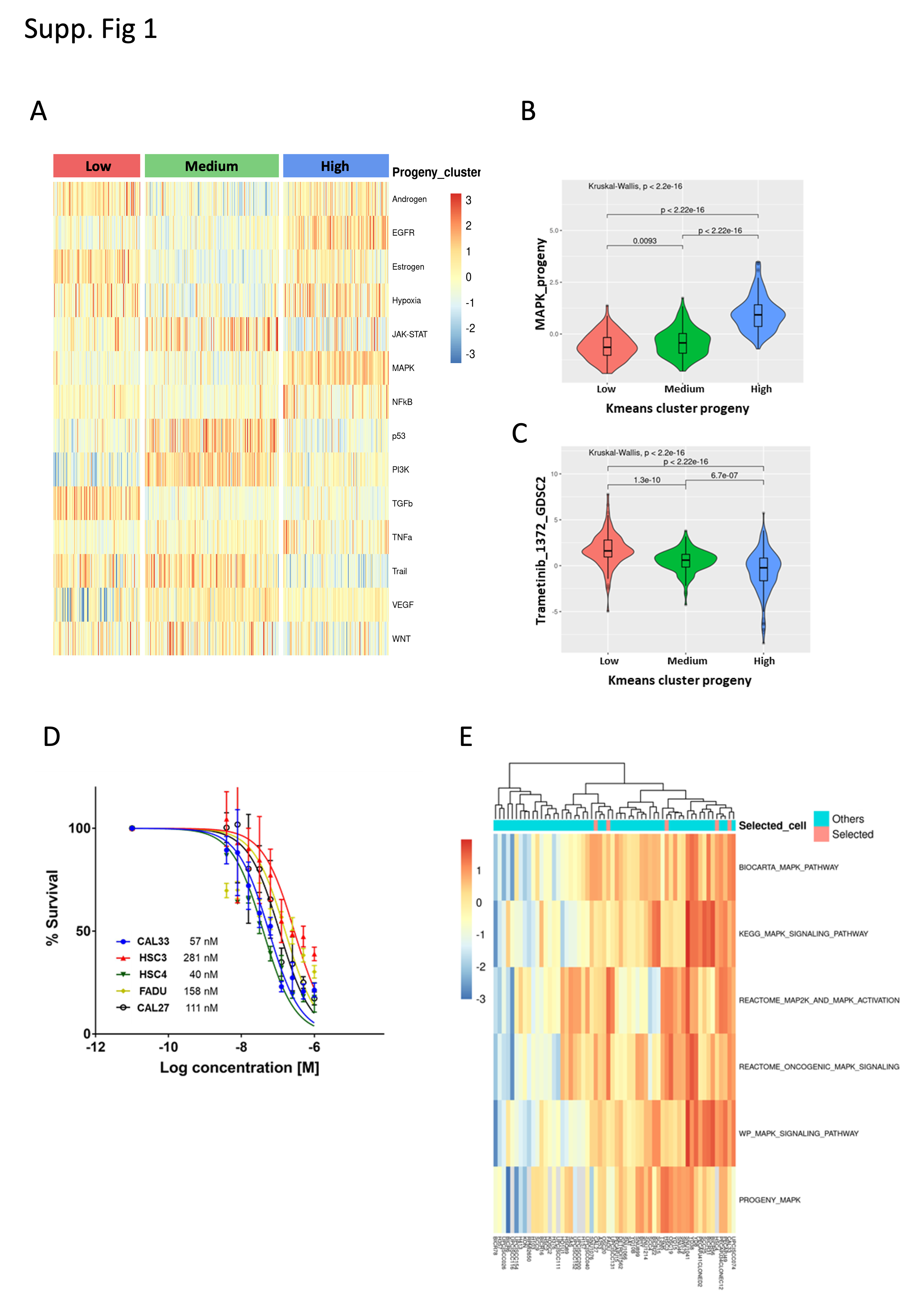

Supplement: Supplementary file 1 — Fig. S1. Sensitivity to trametinib in HNSCC. (A) Heatmap represents the 11 PROGENy pathway scores for primary tumor samples from HPV16‐Negative TCGA‐HNSSC patients, the scores are clustered according to k‐means clustering (k = 3) and clusters are named according to the MAPK activity. (B) Violin plot represents PROGENy MAPK score for the different clusters. (C) Violin plot represents predicted Trametinib sensitivity score calculated using Sanger's Genomics of Drug Sensitivity in Cancer 2 (GDSC2) screening data in previously established MAPK‐activity groups. (D) IC50 values indicating sensitivity to trametinib after 96 h of treatment. Data represent a representative experiment from three independent experiments. Error bars indicate SD. (E) Heatmap shows unsupervised hierarchical clustering of CCLE HNSCC cell lines based on GSVA scores for selected MAPK gene signatures and MAPK PROGENy pathways. (F) High and low exposure of protein array (PathScan® protein array) blots of CAL33 and HSC3 cells treated with 20 nM trametinib for 24 h. (G) Representative immunofluorescent staining of YAP1 (red) and DAPI (blue) in tissue samples (X20, 50 μm) after 25 days of treatment with vehicle or trametinib (0.5 mg/kg). (H) Example of the analysis method and detection threshold for EGFR quantification in QuPath software. Data represent a representative experiment from two independent experiments. [file MOL2-17-2618-s003.zip › FIG S1 A-E.tiff]

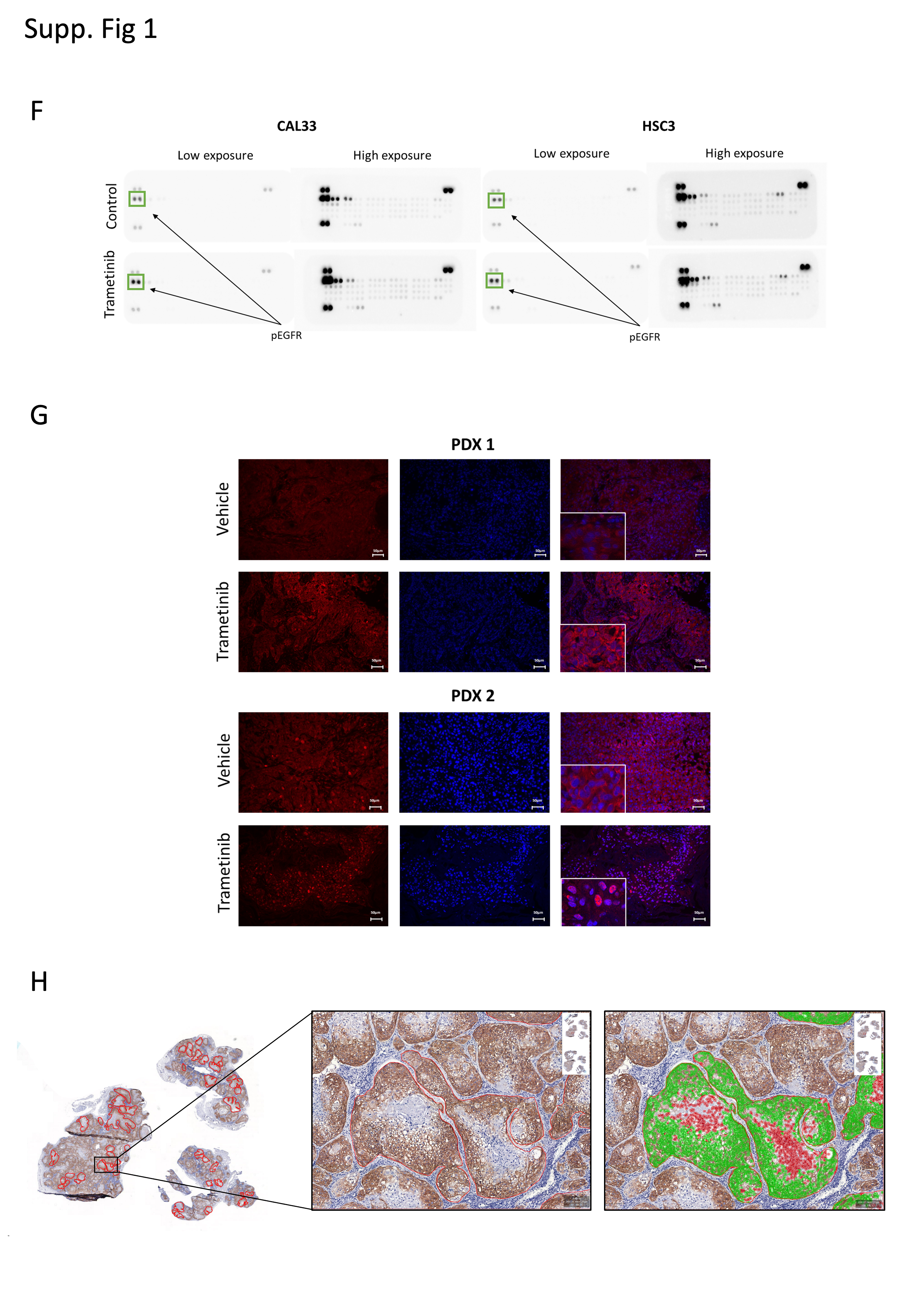

Supplement: Supplementary file 1 — Fig. S1. Sensitivity to trametinib in HNSCC. (A) Heatmap represents the 11 PROGENy pathway scores for primary tumor samples from HPV16‐Negative TCGA‐HNSSC patients, the scores are clustered according to k‐means clustering (k = 3) and clusters are named according to the MAPK activity. (B) Violin plot represents PROGENy MAPK score for the different clusters. (C) Violin plot represents predicted Trametinib sensitivity score calculated using Sanger's Genomics of Drug Sensitivity in Cancer 2 (GDSC2) screening data in previously established MAPK‐activity groups. (D) IC50 values indicating sensitivity to trametinib after 96 h of treatment. Data represent a representative experiment from three independent experiments. Error bars indicate SD. (E) Heatmap shows unsupervised hierarchical clustering of CCLE HNSCC cell lines based on GSVA scores for selected MAPK gene signatures and MAPK PROGENy pathways. (F) High and low exposure of protein array (PathScan® protein array) blots of CAL33 and HSC3 cells treated with 20 nM trametinib for 24 h. (G) Representative immunofluorescent staining of YAP1 (red) and DAPI (blue) in tissue samples (X20, 50 μm) after 25 days of treatment with vehicle or trametinib (0.5 mg/kg). (H) Example of the analysis method and detection threshold for EGFR quantification in QuPath software. Data represent a representative experiment from two independent experiments. [file MOL2-17-2618-s003.zip › FIG S1 F-H.tiff]

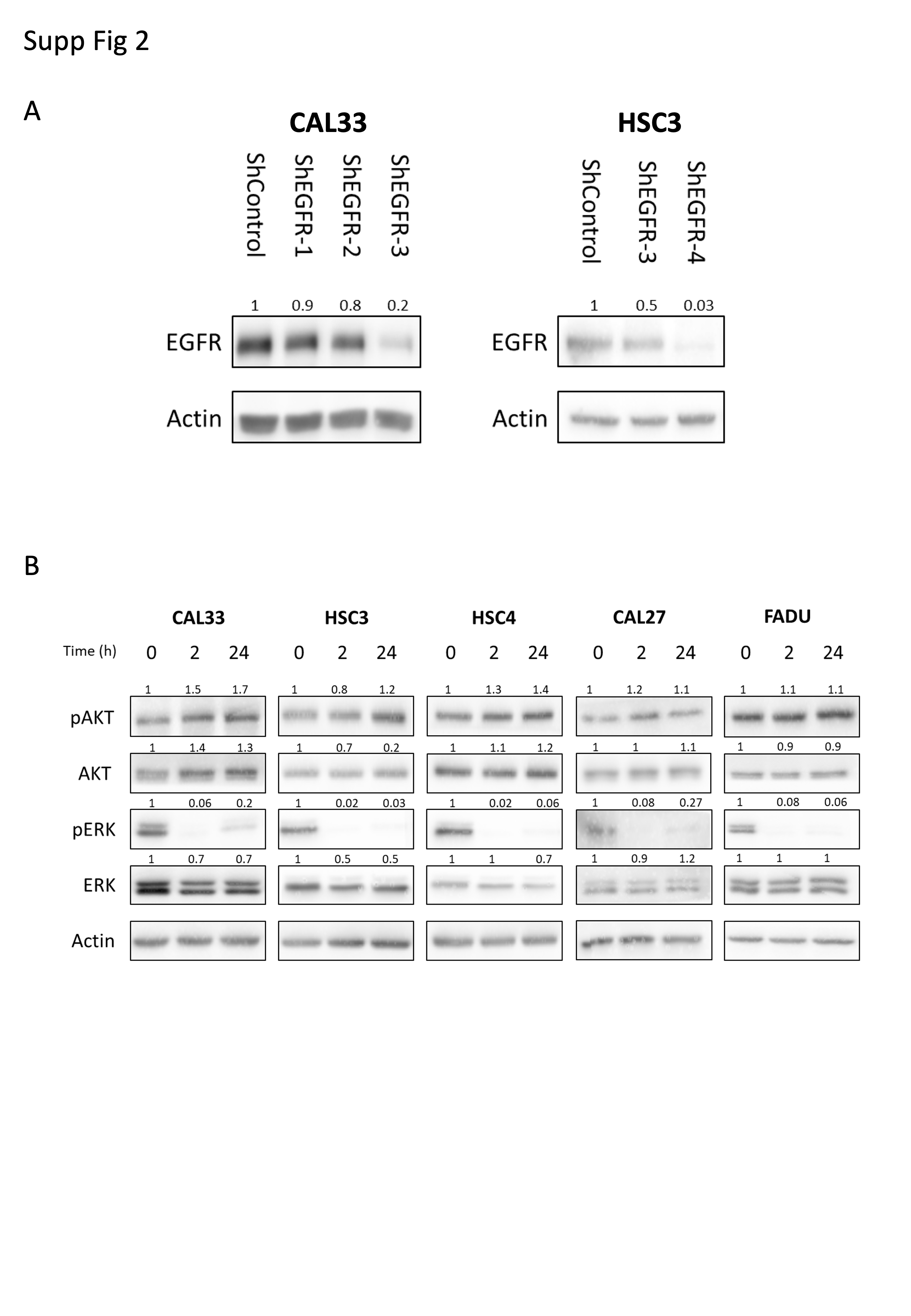

Supplement: Supplementary file 2 — Fig. S2. AKT activation by trametinib treatment. (A) Western blot for Epidermal growth factor receptor (EGFR) protein levels in CAL33 and HSC3 cells infected with control or shEGFR‐1, shEGFR‐2, shEGFR‐3 or shEGFR‐4 PLKO silencing vectors. (B) Western blot for the indicated proteins following 2 and 24 h of treatment with 20 nM trametinib in five human cell lines (CAL33, HSC3, HSC4, FADU, and CAL27). Numbers indicate the fold change in protein level normalized to actin. Data represent a representative experiment from two independent experiments. [file MOL2-17-2618-s002.tiff]

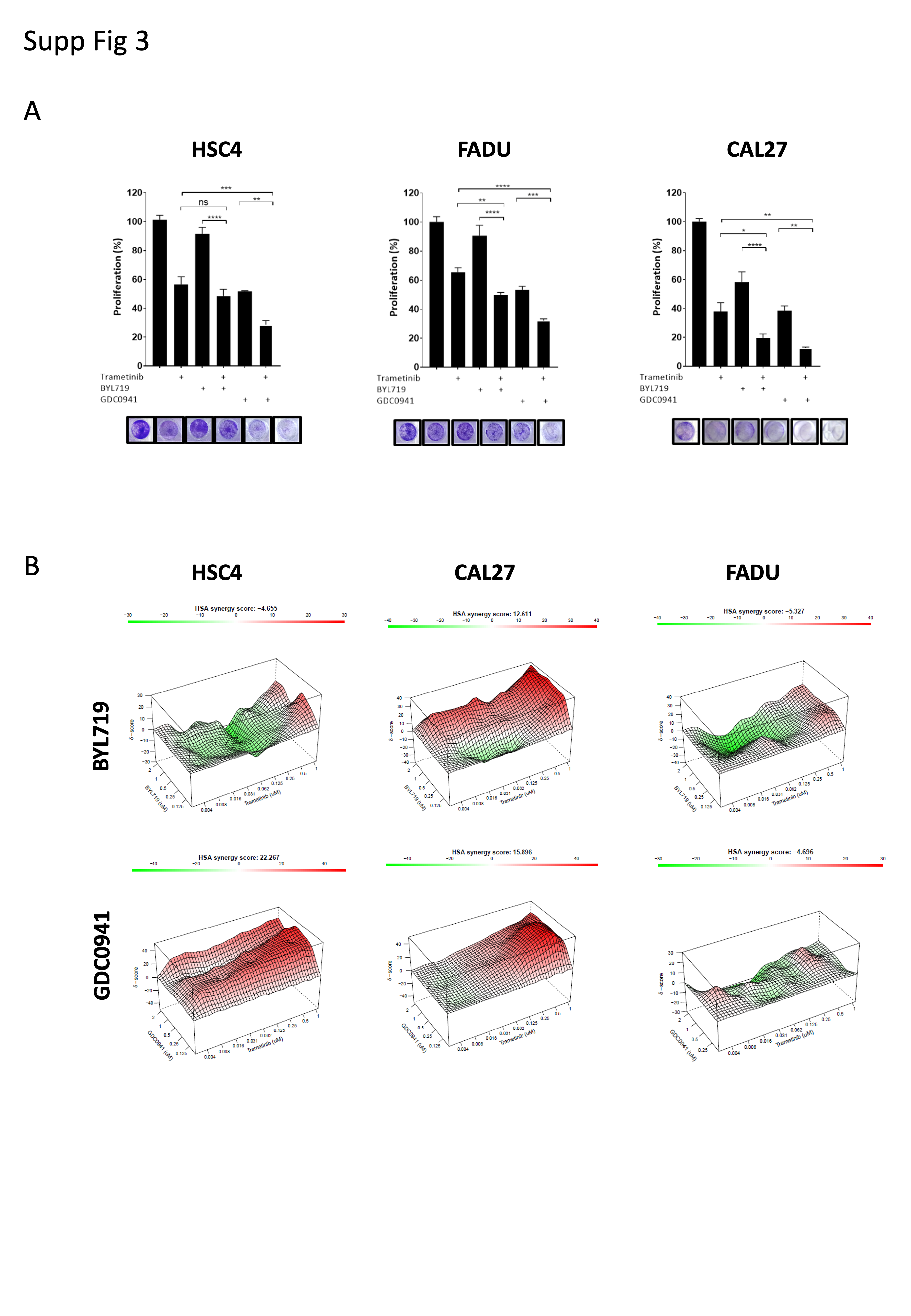

Supplement: Supplementary file 3 — Fig. S3. Efficacy of dual treatment in HNSCC cell lines. (A) 4 Four‐day proliferation assay testing the efficacy of trametinib (20 nM) and of BYL719 (2 μM), or GDC‐0941 (1 μM), alone or in combination, in HSC4, CAL27, and FADU HNSCC cell lines. Data represent a representative experiment from three independent experiments. Error bars indicate SD. Statistical significance was calculated using one‐way ANOVA (ns – not significant, *p < 0.05, **p < 0.01, ***p < 0.001, ****p < 0.0001). (B) Synergy scores and heat map calculated by SynergyFinder for the combination of trametinib with BYL719 or GDC‐0941 in HSC4, CAL27, and FADU HNSCC cell lines. Data represent a representative experiment from three independent experiments. [file MOL2-17-2618-s005.tiff]

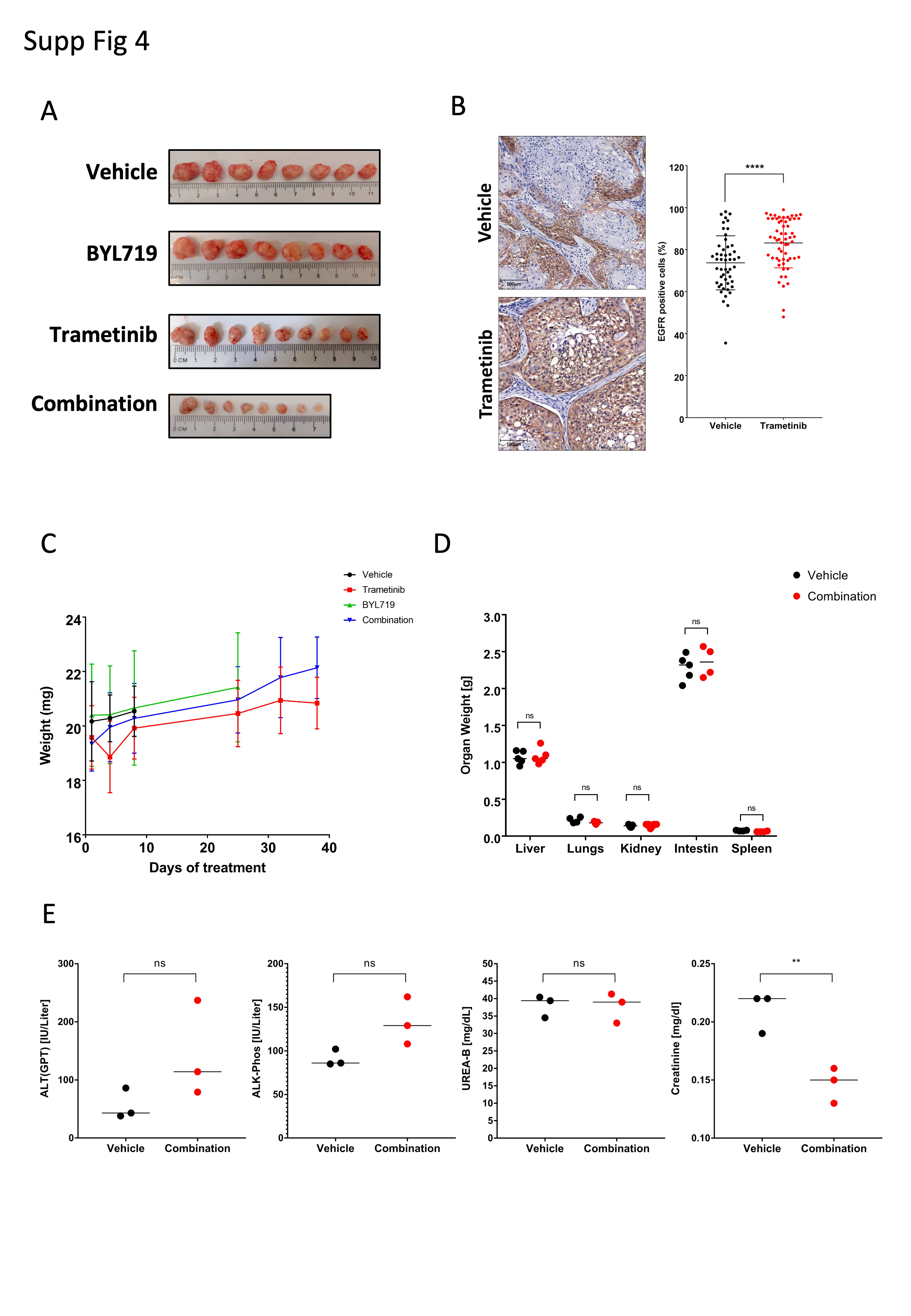

Supplement: Supplementary file 4 — Fig. S4. Efficacy and toxicity of combination therapy. (A) Image of the dissected tumors at end point of the experiment. (B) Representative immunohistochemical staining of epidermal growth factor receptor (EGFR) in tissue samples of PDX‐3 (X20, 100 μm) and quantification of EGFR cells (%, per field) after 20–25 days of treatment with vehicle or trametinib (0.5 mg/kg) (n = 4 tumors and n = 50 analysis fields). Error bars indicate SD. Statistical significance was calculated using the unpaired t‐test (****p < 0.0001). (C) Monitoring of body weight of PDX3‐implanted NSG mice treated with vehicle, trametinib (0.5 mg/kg) by intraperitoneal injection, BYL719 (25 mg/kg) by oral gavage, or a BYL719/trametinib combination treatment for 40 days. Error bars indicate SD. (D) Organ weight of C57BL/6J (WT) mice (n = 5) treated for 7 days with vehicle (corn oil, i.p, and 5% CMC, oral gavage) or trametinib (0.5 mg/kg, i.p) and BYL719 (25 mg/kg, oral gavage). Line indicated median. (E) serum levels of alanine aminotransferase (ALT) and alkaline phosphatase (ALK‐phos), urea and creatinine after 7 days of treatment with vehicle (corn oil, i.p, and 5% CMC, oral gavage) or trametinib (0.5 mg/kg, i.p) and BYL719 (25 mg/kg, oral gavage). Line indicated median. [file MOL2-17-2618-s001.tiff]

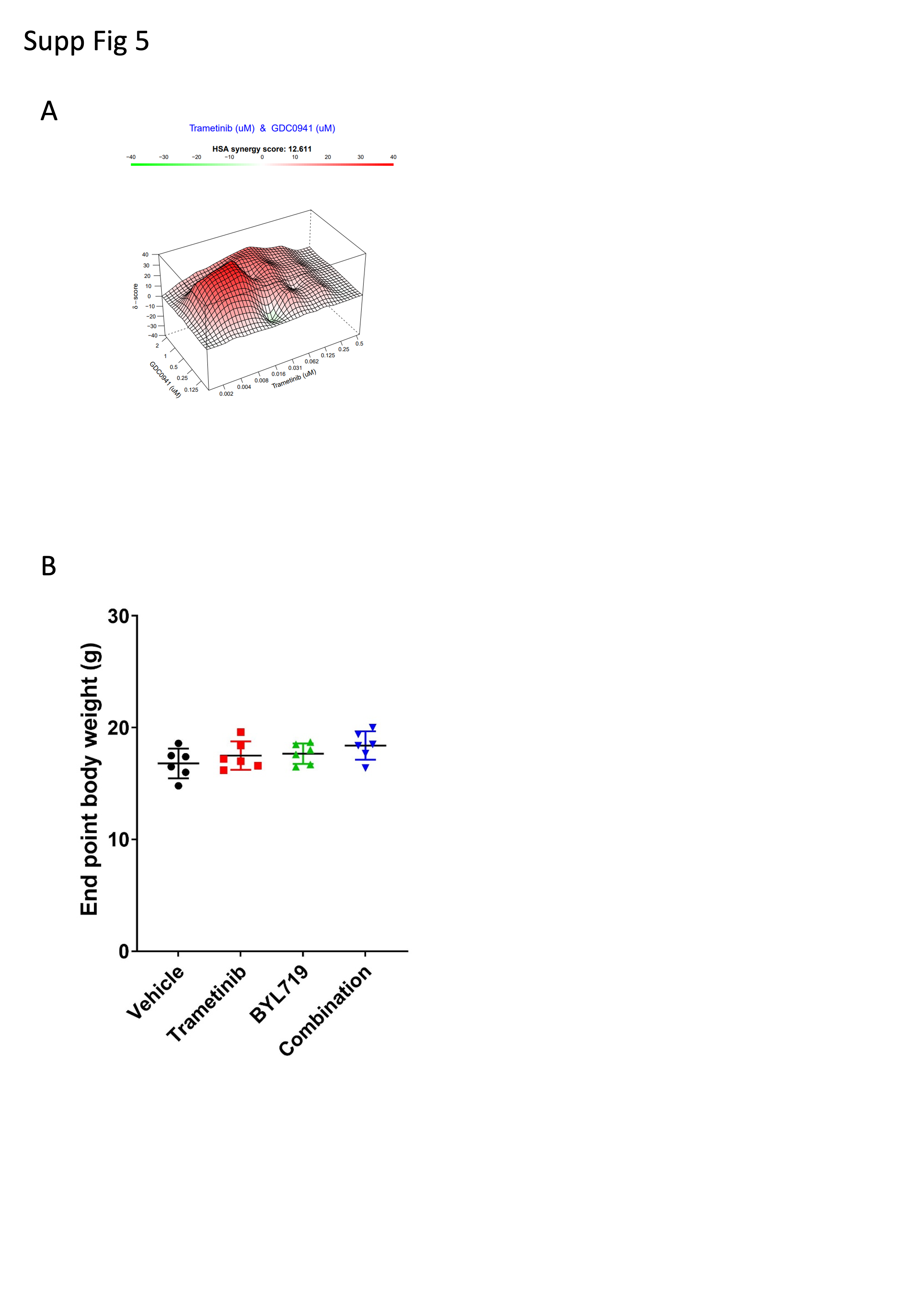

Supplement: Supplementary file 5 — Fig. S5. Efficacy and toxicity in syngeneic HNC murine model. (A) Synergy scores and heatmap calculated by SynergyFinder for the combination of trametinib with GDC‐0941 in a 4NQO‐L murine HNSCC cell line. Data represent a representative experiment from three independent experiments. (B) Body weight of C57BL/6J (WT) mice bearing 4NQO‐L tumors treated with vehicle, trametinib (0.5 mg/kg) by intraperitoneal injection, BYL719 (25 mg/kg) by oral gavage, or with a BYL719/ trametinib combination treatment. Error bars indicate SD. [file MOL2-17-2618-s004.tiff]
